# Audit cycle improves the quality of assistance in the management of stillbirth: A 10 years’ experience

**DOI:** 10.1002/pmf2.70141

**Published:** 2025-10-24

**Authors:** Francesca Monari, Martina Benuzzi, Riccardo Cuoghi Costantini, Gloria Guariglia, Beatrice Melis, Isabella Neri, Antonio La Marca, Fabio Facchinetti, Enrica Perrone, for the Stillbirth Emilia‐Romagna Audit Group

**Affiliations:** ^1^ Obstetrics and Gynecology Unit, Mother‐Infant and Adult Department of Medical and Surgical Sciences University of Modena and Reggio Emilia Modena Italy; ^2^ Mother‐Infant and Adult Department of Medical and Surgical Sciences University of Modena and Reggio Emilia Modena Italy; ^3^ University of Modena and Reggio Emilia Modena Italy; ^4^ Department of Community Care Emilia‐Romagna Health and Welfare Directorate Bologna Italy

**Keywords:** audit cycle, quality of assistance, stillbirth

## Abstract

**Objective:**

Stillbirth represents a tragic event, affecting families as well as caregivers. Evidence suggests that a portion of stillbirths is preventable. The audit cycle is a method to improve healthcare. This study evaluates the effectiveness of this methodology applied within a Perinatal Surveillance System in improving the quality of care and its association with a lower proportion of potentially preventable stillbirths.

**Methods:**

We conducted a prospective evaluation of the quality of assistance and compared two 5‐year periods (2014–2018 and 2019–2023) in the Emilia‐Romagna Region (North of Italy). The audit cycle was implemented, as detailed in the text, in two steps: at local and central levels. In addition to temporal trends in its rate, stillbirth causes and their preventability were evaluated. Population features were also recorded. Univariable logistic regression models were used. Odds ratios (ORs) and corresponding 95% confidence intervals (CIs) were reported.

**Results:**

In the study period, 1041 stillbirths occurred. These stillbirths underwent the same diagnostic work‐up and audit evaluation. The overall stillbirth rate was 0.33%. When comparing the two 5‐year periods, the proportion increased slightly (0.313% vs. 0.354%). We observed a significant reduction in maternal conditions as the primary cause of stillbirth (OR, 0.34; 95% CI, 0.16–0.66) as well as unexplained cases (OR, 0.62; 95% CI, 0.16–0.66) during the second 5‐year period (2019–2023). The quality of assistance also improved over time, with the proportion of inadequate care decreasing from 9.92% in 2014–2018 to 6.71% in 2019–2023 (OR, 0.63; 95% CI, 0.40–0.98).

**Conclusion:**

The audit cycle was associated with fewer preventable and unexplained stillbirths. The overall proportion increased slightly between periods, likely due to changing maternal risk factors, but the audit helped maintain a low rate and improve the quality of care.

## 1 INTRODUCTION

Stillbirth is a devastating event affecting not only parents and their families, but also the caregivers as well as the entire health care system. Stillbirth is a significant public health challenge, even in high‐income countries [1, 2]. In 2021, the United States’ rate of stillbirth (defined as fetal loss at ≥20 weeks’ gestation) was 5.73 per 1000 births—equivalent to 1 in 175 pregnancies—with approximately 21,000 stillbirths occurring annually. In Italy, where a universally offered health care system is in place, the stillbirth has a 50% reduced prevalence (2.4 per 1000 live births [3], similar to other European countries such as Sweden, Norway, Poland, Spain, Estonia, and Lithuania [2].

Despite advances in treatments and diagnostic techniques, stillbirth remains one of the most complex and challenging issues in perinatal medicine. Evidence suggests that many stillbirths are preventable. In high‐income countries, suboptimal care has been estimated to contribute to 60% of antepartum‐ and up to 80% of intrapartum‐related deaths, according to findings from national audit systems such as the UK's CESDI and other studies [4]. The proportion of avoidable stillbirths depends on the baseline stillbirth rate and may vary significantly between countries. Key risk factors for stillbirth include intrauterine growth restriction (IUGR), maternal medical conditions (such as obesity, diabetes, and hypertension), and placental disfunctions, which are often inadequately managed [4, 5]. The management of these cases involves a series of complex clinical decisions and requires providing adequate care to women, both from a medical and a psychosocial perspective. In this context, the implementation of the clinical audit systems has gained attention as a tool to monitor and improve the quality of assistance, identifying potential errors and promoting reflection on diagnostic and therapeutic pathways. In the Netherlands, the rapid nationwide implementation of perinatal audit was associated with a reduction in term perinatal mortality, suggesting its potential to improve the quality of care [6].

Worldwide several health care facilities have adopted Safer Baby Bundle protocols [1, 7] to prevent and reduce stillbirth occurrence. The United Kingdom introduced the *Saving Babies Lives* care bundle in 2015 [1] and the Australian Senate prioritized stillbirth prevention by launching the *Safer Baby Bundle* [7]. Moreover, a study [8] demonstrated that the audit system for the management of stillbirth has the potential to reduce variability in the management, ensuring a more timely, appropriate, and empathetic response for the women involved. This system has the aim of improving clinical outcomes and optimizing care protocols, and, through a systematic and critical review process of cases and the collection of data, promotes the identification of areas for improvement and the standardization of clinical practices.

In Emilia‐Romagna, an Italian region with an advanced health care system, a Perinatal Stillbirth Surveillance System has been in place since 2014. This surveillance is crucial for increasing awareness of stillbirth as a public health issue, improving diagnostic and care practices for stillbirth cases, enhancing parental support services, strengthening data collection, and designing targeted and feasible preventive interventions.

The primary objective of this study is to evaluate the effectiveness of the audit cycle (Plan, Do, Check, Act) application within the Perinatal Stillbirth Surveillance System throughout a 10‐year period, specifically assessing quality of assistance and changes in preventable deaths.

## 2 MATERIAL AND METHODS

Stillbirth is defined as fetal death occurring at 22 weeks of gestation or greater (or birthweight of at least 500 g if the gestational age is unknown) according to WHO definition [9].

We included in this area‐based study all stillbirths that occurred in Emilia‐Romagna between 2014 and 2023. This study received IRB approval. Informed consent for diagnostic work‐up was not required, as the investigations following stillbirth are mandatory by law in Italy (D.M. 7/2014 and D.P.C. 170/99). *Therefore, participation in the audit process did not require external incentives, as the systematic review of all cases is embedded in clinical governance*.

Information was stored anonymously in a secure electronic database. Patient and fetus privacy were preserved and respected during data collection and analysis. To ensure confidentiality, data cells with fewer than three cases were labelled as “<3.”

### 2.1 The audit system

The surveillance system investigates stillbirth through a program based on the audit cycle model. The cycle starts at every birth center by identifying cases, collecting systematic information from either clinical records or directly from the mother, then providing that each case was submitted to the protocol of mandatory examinations, as agreed by the group [10].

Briefly, information includes sociodemographic data of the mother as well as her medical and obstetric history. Fetal health indicators such as fetal movements, weight at birth, and placenta weight were documented, alongside information on any pregnancy complication such as intrauterine growth restriction, cord prolapse, or placental abruption. Laboratory tests and genetic investigation, placenta histology and fetal autopsy, as well as ultrasound scans were also part of the collection. Placental pathology was performed in more than 95% of cases and fetal autopsy in about 90%, both carried out by dedicated perinatal pathologists. Placental examinations were uniformly evaluated according to the Amsterdam consensus guidelines, ensuring consistent reporting across centers. Maternal health behaviors, including smoking, alcohol use, and drug consumption, were captured.

**FIGURE 1:**
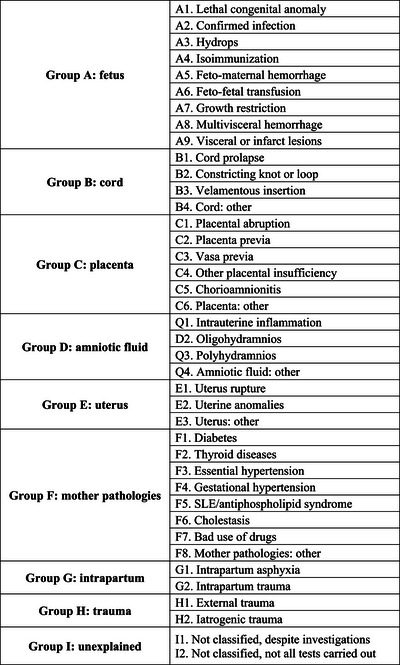
Primary cause of death according to the ReCoDe classification [11].

Once data are available, a county group (Local Audit Group), composed of at least an obstetrician, a neonatologist, and a pathologist from the clinical teams (led by a local coordinator), critically analyzes data and, after a thorough discussion, assigns a primary cause of death, also describing the circumstances. Causes of stillbirth were then categorized by using the ReCoDe classification [11] (Figure 1). The primary cause of death was assigned collegially by the Local Audit Group using the ReCoDe classification, based on the complete diagnostic work‐up (clinical records, laboratory tests, placental histology, and fetal autopsy). In cases without agreement, the Central Audit Group discussed the case and reached the final decision. As the ReCoDe classification records the relevant condition at death rather than a proven cause, the assignment was made only when the condition was considered by consensus to plausibly contribute to the fatal outcome, in the context of all available clinical, pathological, and autopsy information, and not when judged as an incidental finding. To ensure consistency over time, all cases were classified using the ReCoDe system and discussed collegially within Local and Central Audit Groups. In complex cases with multiple findings, the primary cause was assigned only when judged plausibly related to the death, while incidental findings were not considered.

The Local Audit Group also classifies the quality of assistance according to the Confidential Enquiry into stillbirths and Deaths in Infancy (CESDI) rating [12] where: 0 = no substandard care; 1 = substandard care but changing the management would not have impacted the outcome; 2 = substandard care, with a potential for improved outcomes with different management, and 3 = substandard care, where alternative management would likely have led to a better outcome. For statistical purposes, these four scores were grouped into two main categories: specifically considering the adequate care if 0 or 1 were assigned, and inadequate care (potentially avoidable death) if 2 or 3 were assigned. To minimize subjectivity, each case was first assessed by the Local Audit Group and, when necessary, re‐evaluated by the Central Audit Group. The use of standardized CESDI criteria combined with this two‐step collegial review ensured consistency and reproducibility in attributing substandard care.

Inadequate management typically includes delayed or missed diagnosis of fetal growth restriction, or if diagnosed, inadequate management of the timing of delivery, insufficient monitoring in pregnancies complicated by diabetes or hypertension, and failure to escalate care when abnormal findings (e.g., abnormal Doppler velocimetry, reduced fetal movements, or borderline amniotic fluid levels) are present. These situations often reappear across multiple cases, indicating the need for earlier identification of risk factors and stricter adherence to clinical protocols.

A sheet containing a single‐case summary of the local discussion was then transmitted to the regional commission (Central Audit Group) together with details of those cases lacking agreement for which a discussion is required with the Central Audit Group. Such a group is composed of representatives of the local groups (eight for the entire area) and includes multidisciplinary experts, such as feto‐placental pathologists, microbiologists, geneticists, methodologists, etc. The Central Audit Group, which meets 3 times/year, sends back to the Local Audit Group the final categorization of the pertinent cases as well as the request for major details when the attribution of the cause of death/quality of assistance is not reached. This structured two‐step evaluation (Local and Central Audit Groups) based on the CESDI criteria ensured objective and reproducible assessments, while also allowing us to identify the most frequent sources of substandard care, mainly delayed recognition or inadequate timing of delivery of fetal growth restriction, inadequate monitoring of maternal conditions, and insufficient response to abnormal findings.

### 2.2 Definitions and statistical analysis

A prepregnancy body mass index (BMI) ≥25 kg/m^2^ was classified as overweight. Gestational weight gain and neonatal anthropometry were assessed using the Institute of Medicine's guidelines and the Italian Neonatal Study chart, respectively [13, 14]. Small for gestational age (SGA), adequate for gestational age (AGA), and large for gestational age (LGA) were defined based on the neonatal birth weight percentile: <10%, between 10% and 90%, or >90%, respectively [15]. Descriptive statistics were computed for each variable of interest, according to its nature: categorical variables were reported using absolute frequencies and percentages, whereas numerical variables were reported as mean and standard deviation, or median and interquartile range. To investigate temporal trends in stillbirth causes and the association between quality of assistance and specific etiologies, univariable logistic regression models were used. The results obtained were reported in terms of odds ratios (ORs) and their corresponding 95% confidence intervals (CIs). Statistical significance of OR was determined using the Wald test. Fisher's exact test was also used to assess the associations between the quality of assistance and the primary causes of fetal death, as the resulting contingency table included cells with very low observed frequencies. The significance level alpha was set at 0.05 for all analyses. No imputation was applied, and analyses were conducted using complete case data. All analyses were carried out using R statistical software version 4.3.2 (The R Foundation for Statistical Computing, https://www.R‐project.org/).

## 3 RESULTS

During the study period, 1041 stillbirths occurred in Emilia‐Romagna, every one of them underwent an audit evaluation. The overall stillbirth rate was 3.3 per 1000 births, while annual rate changes are reported in Table 1. When comparing the two 5‐year periods, the overall stillbirth proportion slightly increased (0.313% in 2014–2018 vs. 0.354% in 2019–2023), a trend that is further explored in Section 4.

**TABLE 1:** Overall SB (≥22 weeks) rate during the study period.

	Livebirths (*N* = 324,883)		SBs (*N* = 1079)		OR	95% CI		*p* value
	*N*	Perc (%)	*N*	Perc (%)				
2014	36,740	99.69	113	0.31	Reference			
2015	35,778	99.70	109	0.30	0.99	0.76	1.29	0.944
2016	34,694	99.70	103	0.30	0.97	0.74	1.26	0.795
2017	33,380	99.66	115	0.34	1.12	0.86	1.45	0.392
2018	32,854	99.69	103	0.31	1.02	0.78	1.33	0.888
2019	31,499	99.63	117	0.37	1.21	0.93	1.56	0.153
2020	30,230	99.64	110	0.36	1.18	0.91	1.54	0.210
2021	30,272	99.66	102	0.34	1.10	0.84	1.43	0.505
2022	30,428	99.70	93	0.30	0.99	0.75	1.31	0.964
2023	29,008	99.61	114	0.39	1.28	0.98	1.66	0.065

Abbreviations: CI, confidence interval; OR, odds ratio; SB, stillbirth.

Table 2 shows a statistically significant reduction in maternal conditions as the primary cause of stillbirth during the second 5‐year period (2019–2023) (*p* = 0.002). Moreover, we also noticed a significant reduction in the category of “unexplained cases of stillbirth despite investigations” (I1) (*p* = 0.008).

**TABLE 2:** Differences in primary condition associated with SB^a^ during the study periods (2019–2023 vs. 2014–2018).

Primary condition	Study periods	OR	95% CI		*p* value
Group A: fetus	2019–2023 vs. 2014–2018	1.29	0.96	1.74	0.094
Group B: umbilical cord	2019–2023 vs. 2014–2018	1.08	0.75	1.54	0.681
Group C: placenta	2019–2023 vs. 2014–2018	1.14	0.89	1.46	0.303
Group D: amniotic fluid	2019–2023 vs. 2014–2018	1.07	0.13	8.92	0.948
Group E: uterus	2019–2023 vs. 2014–2018	0.26	0.04	1.06	0.093
Group F: mother	2019–2023 vs. 2014–2018	**0.34**	**0.16**	**0.66**	**0.002**
Group G: intrapartum	2019–2023 vs. 2014–2018	1.20	0.46	3.23	0.705
Group H: trauma	2019–2023 vs. 2014–2018	1.07	0.04	27.03	0.963
Group I: unexplained	2019–2023 vs. 2014–2018	0.80	0.59	1.09	0.156
I1 *Not classified, despite investigations*	2019–2023 vs. 2014–2018	**0.62**	**0.44**	**0.88**	**0.008**
I2 *Not classified, not all tests carried out*	2019–2023 vs. 2014–2018	1.64	0.92	2.96	0.095

Abbreviations: CI, confidence interval; OR, odds ratio; SB, stillbirth.

^a^ According to the ReCoDe classification.

Bold values show all the numbers that refer to that specific significance values.

Overall, stillbirths defined as associated with adequate care were 954, while inadequate care was determined in the remaining 87 (8.36%). The quality of assistance was differently associated with the primary causes of death (Table 3). The inadequacies of care were more likely to be present in cases of IUGR (A7), other forms of placental insufficiency (C4), gestational or pregestational diabetes (F1), uterine rupture (E1), chronic hypertension (F3), and gestational hypertension (F4).

**TABLE 3:** Relationship between causes of death and QoA.^a^

	QoA								
	Adequate (*N* = 942)		Inadequate (*N* = 87)		Risk inadequate vs. adequate QoA				
Primary condition	Freq (*n*)	Perc (%)	Freq (*n*)	Perc (%)	Odds ratio	95% CI		*p* value	Fisher's test *p* value
**A1**	**51**	**5.41**	**<3**	**0.00**	**0.95**	**0.90**	**0.99**	**0.026**	**0.017**
A2	44	4.67	4	4.60	1.00	0.95	1.05	0.975	1.000
A3	4	0.42	<3	0.00	1.00	0.98	1.01	0.543	1.000
A4	<3	0.21	<3	0.00	1.00	0.99	1.01	0.667	1.000
A5	21	2.23	<3	2.30	1.00	0.97	1.03	0.967	0.708
A6	14	1.49	<3	2.30	1.01	0.98	1.04	0.558	0.635
**A7**	**59**	**6.26**	**13**	**14.94**	**1.09**	**1.03**	**1.15**	**0.002**	**0.007**
B1	<3	0.21	<3	0.00	1.00	0.99	1.01	0.667	1.000
**B2**	**72**	**7.64**	**<3**	**0.00**	**0.93**	**0.88**	**0.98**	**0.007**	**0.002**
B3	4	0.42	<3	0.00	1.00	0.98	1.01	0.543	1.000
B4	57	6.05	3	3.45	0.97	0.93	1.03	0.322	0.470
**C1**	**128**	**13.59**	**5**	**5.75**	**0.92**	**0.86**	**0.99**	**0.037**	**0.030**
**C4**	**160**	**16.99**	**25**	**28.74**	**1.12**	**1.03**	**1.22**	**0.006**	**0.008**
**C5**	**66**	**7.01**	**<3**	**1.15**	**0.94**	**0.89**	**0.99**	**0.034**	**0.037**
C6	23	2.44	<3	0.00	0.98	0.94	1.01	0.141	0.247
D1	4	0.42	<3	0.00	1.00	0.98	1.01	0.543	1.000
**E1**	**6**	**0.64**	**3**	**3.45**	**1.03**	**1.01**	**1.05**	**0.007**	**0.027**
E3	<3	0.00	<3	1.15	1.01	1.00	1.02	0.001	0.088
**F1**	**12**	**1.27**	**8**	**9.20**	**1.08**	**1.05**	**1.12**	**0.000**	**0.000**
F2	4	0.42	<3	0.00	1.00	0.98	1.01	0.543	1.000
**F3**	**<3**	**0.11**	<3	**2.30**	**1.02**	**1.01**	**1.03**	**0.000**	**0.022**
**F4**	**9**	**0.96**	**4**	**4.60**	**1.04**	**1.01**	**1.06**	**0.004**	**0.016**
F5	<3	0.00	<3	1.15	1.01	1.00	1.02	0.001	0.088
F8	<3	0.11	<3	0.00	1.00	0.99	1.01	0.761	1.000
G1	15	1.59	<3	2.30	1.01	0.98	1.04	0.621	0.657
H1	<3	0.11	<3	0.00	1.00	0.99	1.01	0.761	1.000
H2	<3	0.11	<3	0.00	1.00	0.99	1.01	0.761	1.000
I	<3	0.11	<3	0.00	1.00	0.99	1.01	0.761	1.000
**I1**	**142**	**15.07**	**6**	**6.90**	**0.92**	**0.85**	**0.99**	**0.038**	**0.037**
I2	38	4.03	5	5.75	1.02	0.97	1.06	0.445	0.367

Abbreviations: CI, confidence interval; OR, odds ratio; QoA, quality of assistance.

^a^ According to the ReCoDe classification in Figure 1.

Bold values show all the numbers that refer to that specific significance values.

Conversely, cases of stillbirth due to cord loops or knots (B2), placental abruption (C1), chorioamnionitis (C5), lethal congenital anomaly (A1), and unexplained factors are less likely to receive inadequate care.

To assess temporal changes in the population features, hence possible shifts in associated risk factors, Table 4 reports the most important maternal risk factors for stillbirth during the first and last 5 years of the study period.

**TABLE 4:** Trends in SB risk factors over time.

	2014–2018 (*N* = 173,989)		2019–2023 (*N* = 151,973)		
Variable	*N*	%	*N*	%	*p* value
Maternal Age					
>35 years	49,535/173,794	28.5	43,288/151,819	28.5	0.949
Maternal education					
High	56,794	32.7	55,543	36.6	<0.001
Medium	73,514	42.3	61,672	40.6	
Low	43,634	25.1	34,685	22.8	
Smoking					
Yes	11,335/171,996	6.6	8713/148,843	5.8	< 0.001
BMI					
Normal weight	110,369	65.2	94,717	62.3	<0.001
Underweight	12,551	7.4	10,090	6.6	
Overweight	31,569	18.6	31,073	20.4	
Type I obesity	10,505	6.2	11,336	7.5	
Type II–III obesity	4285	2.5	4717	3.1	
IVF					
Yes	5380/171,729	3.1	5375/151,095	3.6	<0.001
Previous delivery					
Yes	85,376/173,988	49.1	75,399/151,960	49.5	0.006
GWG					
Adequate	46,870	38.9	54,737	38.6	<0.001
Inadequate (high)	28,817	23.9	32,633	23.0	
Inadequate (low)	44,674	37.1	54,429	38.4	
Area of origin					
Italy	118,124	68.0	103,993	68.4	<0.001
Asia	3927	2.3	2617	1.7	
Eastern Europe	22,912	13.2	19,175	12.6	
Indian subcontinent	6318	3.6	6580	4.3	
Latin America	2101	1.2	1853	1.2	
North Africa	12,896	7.4	10,250	6.7	
Sub‐Saharan Africa	6377	3.7	6499	4.3	
Western Europe	896	0.5	779	0.5	

Abbreviations: BMI, body mass index; IVF, in vitro fertilization; GWG, gestational weight gain; SB, stillbirth.

The quality of assistance was significantly improved over time, with the proportion of inadequate care decreasing from 9.93% in 2014–2018 (53/534 cases) to 6.71% in 2019–2023 (34/507 cases). This association was statistically significant (OR, 0.63; 95% CI, 0.40–0.98, *p* value 0.041).

## 4 DISCUSSION

Although we work in a high‐resource setting, with one of the lowest rates of stillbirth in European countries, during the first 5 years of observation, almost 1 in 10 cases was judged by professionals as potentially avoidable. During the audit cycle, the proportion was significantly reduced by approximately one‐third. This association suggests that further improvements are still possible, although causality cannot be established. Indeed, this extrapolation should be interpreted with caution, as the proportion of preventable cases depends on the baseline stillbirth rate and on the advancement of the health care system. In addition, no relevant changes in definitions, classification (ReCoDe), or regional guidelines occurred during the study period; the reduction in maternal disease–related stillbirths is therefore more likely related to improved ascertainment and management promoted by the audit process. Globally, there are about 2.6 million fetal demises each year, and 2% of these (approximately 52,000) occur in high‐resource countries [8, 16]. This means that, if we speculate that 6.71% of stillbirths were potentially avoidable, around 3490 cases per year in developed countries could be potentially preventable through improved care quality. However, this estimate should be interpreted with caution, as the proportion of preventable cases depends strongly on the baseline stillbirth rate (the potential for improvement) and on how advanced the health care system is in each context.

Our results show that the improvement in the quality of assistance was concomitantly associated with a reduction of maternal disorders (such as diabetes or hypertension) recognized as the cause of intrauterine death and unexplained causes. Although no specific programs have been implemented, this suggests that discussions within the audit groups allowed more appropriate management of such disorders. The finding is even more important if one considers that obesity at delivery increased during the last years, thus directly increasing the attributable population risk [17].

However, our data indicated that inadequate assistance is significantly identified in cases of stillbirth related to IUGR, confirming this condition as a known and preventable risk factor for fetal demise [18]. In addition to restriction of growth, there are further risk factors where inadequacy of care has been identified, that is, diabetes, hypertension, and placental insufficiency, all conditions where appropriate management would prevent death [8]. These conditions (e.g., A7, C4, F1, F3, F4, E1) warrant increased clinical attention, as enhanced care in these scenarios may reduce the avoidable death. Indeed, this should raise our attention, namely to the first trimester of pregnancy, when, at the first women's approach, family and maternal history as well as simple examinations could indicate customized prophylaxes [19].

On the other hand, the observation that deaths related to cord knots/loops, chorioamnionitis, or lethal congenital anomaly are associated with adequate care indicates the value of perinatal medicine in the primary care, while likely reflects the actual inability to avoid such events. Indeed, a previous study states that, among the fetal demises with a recognized cause, almost a quarter remain unpreventable [20]. In this respect, it should be noticed how, also in our series, one out of six cases remains unexplained, partly due to a lack of complete work‐up.

### 4.1 Comparison with international experiences

The United Kingdom introduced the *Saving Babies’ Lives* care bundle in 2015. This initiative targets four critical areas: smoking cessation, IUGR diagnosis and management, awareness of reduced fetal movements, and management of intrapartum hypoxia. Implementing the *Saving Babies’ Lives* care bundle costs an additional £140 for each pregnancy but has demonstrated promising results in reducing stillbirths and improving outcomes [1]. Similarly, the Australian Senate prioritized stillbirth prevention by launching the *Safer Baby Bundle* in 2019. It includes strategies such as smoking cessation, screening and management of intrauterine growth restriction, raising awareness of reduced fetal movements, promoting safe maternal sleeping positions, and optimizing delivery timing for high‐risk pregnancies, saving at least 150 newborns annually [7].

In Emilia‐Romagna, through the audit cycle, we have done something different, including directly involved hundreds of caregivers, namely obstetricians, midwives, and neonatologists, through voluntary participation in the absence of any specific reward. The main steps were: (1) the stimulation toward the application of the diagnostic protocol and (2) the punctual discussion of every case, including the rating of the quality of assistance. As a result, unexplained cases of stillbirth despite investigations (I1) significantly reduced, together with a decrease in avoidable death. These findings suggest that the Regional Audit System may have improved registration and facilitated the definition of causes of death, although this cannot be considered definitive proof of causality [10]. For international comparisons, the focus is on late stillbirths (fetal loss at ≥28 weeks’ gestation). The late stillbirth rate during last 10 years remains stable in the Emilia‐Romagna Region (range 2.4–3.0 per 1000 births data not shown) and this rate is one of the lower rates in developed countries [21]; indeed, as highlighted by recent Euro‐Peristat data, most European countries have continued to reduce stillbirth rates to very low levels, below 3 per 1000 births [2].

Despite the reduction in inadequate obstetric care over recent years, a corresponding decline in stillbirth rates was not observed. This small increase in the overall proportion of stillbirth may be attributable to a concurrent rise in maternal risk factors, both modifiable—such as obesity ^17^and non‐modifiable, including the increasing prevalence of foreign‐born women and the use of assisted reproductive technologies such as in vitro fertilization (IVF) [3]. Foreign‐born women exhibit a higher risk of stillbirth, notably those migrating from the Indian subcontinent and Sub‐Saharan Africa, as previously documented in previous studies [22, 23]. Interestingly, such upward trend in maternal risk factors associated with stillbirth is not confined to our region but being also reported in other countries [24]. Although complete investigations substantially reduce the proportion of unexplained stillbirths, they are unlikely to identify a cause in all cases. In our cohort, the unexplained category decreased significantly after the audit, yet about one in six cases remained without a definitive attribution. This reflects both occasional incomplete work‐ups and the fact that some lethal mechanisms (e.g., transient hypoxia, primary arrhythmias) may not leave specific postmortem signs, while other findings can be incidental. Thus, even with comprehensive investigations, a small residual of unexplained deaths is inevitable.

### 4.2 Strengths and limitations of our study

There are several strengths of our study. It is area‐based, involves every birth center, covers a long period of time, and has a large sample size. Moreover, the use of standardized diagnostic protocols ensured a homogeneous comparison between cases, while the multidisciplinary and collegial discussion of stillbirths allowed for a more accurate evaluation of causes of death. The uniform adoption of the Amsterdam consensus criteria by all perinatal pathologists in our region further strengthened the reliability and comparability of placental pathology evaluations. Finally, discussion of the case in a multidisciplinary group at local and regional levels, involving professionals not directly involved in the care of the individual case, reduces the risk of altering the attribution of the quality of care.

However, there are also some limitations. Although in a small percentage, the presence of missing or incomplete data made it difficult to precisely determine the cause of fetal demise in some situations. Finally, while the overall stillbirth proportion slightly increased between the two study periods, this likely reflects demographic and maternal risk changes rather than the audit process itself.

## 5 CONCLUSION

The implementation of the audit cycle has proven to be feasible and a valuable tool in our Italian experience for enhancing clinicians' knowledge and expertise regarding stillbirth. Although the audit cycle improved the quality of care and reduced preventable and unexplained stillbirths, the overall stillbirth proportion showed a slight increase between the two periods, most likely reflecting demographic and epidemiological changes in the maternal population and risk factors. Therefore, the audit should be interpreted primarily as a process of learning and quality improvement rather than as a direct causal intervention on stillbirth incidence.

## AUTHOR CONTRIBUTIONS

All authors meet the ICMJE authorship criteria, having made substantial contributions to the conception and design, acquisition of data, or analysis and interpretation of data, and participated in drafting or critically revising the manuscript. Each author has approved the final version and agrees to be accountable for all aspects of the work.

## CONFLICT OF INTEREST STATEMENT

The authors declare no conflicts of interest.

## FUNDING INFORMATION

This research did not receive any specific grant from funding agencies in the public, commercial, or not‐for‐profit sectors.

## ETHIC STATEMENT

This study received the IRB approval (AOU Protocol 0028481/21−September 22, 2021, “Comitato Etico Vasta Area Emilia Nord, AVEN”). Informed consent for diagnostic work‐up was not required, as the investigations following stillbirth are mandatory by law in Italy (D.M. 7/2014 and D.P.C. 170/99). Information was stored anonymously in a secure electronic database. Patient and fetus privacy were preserved and respected during data collection and analysis. To ensure confidentiality, data cells with fewer than three cases were labelled as “<3”.

## PERMISSION TO REPRODUCE MATERIAL FROM OTHER SOURCES

This manuscript does not contain any copyrighted material from other sources.

## Data Availability

The data supporting the findings of this study are available from the corresponding author upon reasonable request.
